# Increase of organic solvent tolerance of *Escherichia coli* by the deletion of two regulator genes, *fadR* and *marR*

**DOI:** 10.1007/s00253-012-4463-8

**Published:** 2012-10-10

**Authors:** Hye Yun Oh, Jae Ok Lee, Ok Bin Kim

**Affiliations:** Department of Life Science, Ewha Womans University, 120-750 Seoul, South Korea

**Keywords:** Organic solvent tolerances, Biofuel, *fadR*, *marR*, Saturated fatty acid, MDR

## Abstract

The improvement of bacterial tolerance to organic solvents is a main prerequisite for the microbial production of biofuels which are toxic to cells. For targeted genetic engineering of *Escherichia coli* to increase organic solvent tolerances (OSTs), we selected and investigated a total of 12 genes that participate in relevant mechanisms to tolerance. In a spot assay of 12 knockout mutants with *n*-hexane and cyclohexane, the genes *fadR* and *marR* were finally selected as the two key genes for engineering. Fatty acid degradation regulon (FadR) regulates the biosynthesis and degradation of fatty acids coordinately, and the multiple antibiotic resistance repressor (MarR) is the repressor of the global regulator MarA for multidrug resistance. In the competitive growth assay, the Δ*marR* mutant became dominant when the pooled culture of 11 knockout mutants was cultivated successively in the presence of organic solvent. The increased OSTs in the Δ*marR* and Δ*fadR* mutants were confirmed by a growth experiment and a viability test. The even more highly enhanced OSTs in the *ΔfadR ΔmarR* double mutant were shown compared with the two single mutants. Cellular fatty acid analysis showed that the high ratio of saturated fatty acids to unsaturated fatty acids plays a crucial role in OSTs. Furthermore, the intracellular accumulation of OST strains was significantly decreased compared with the wild-type strain.

## Introduction

The production of biofuels which replace fossil fuels like gasoline and diesel has been actively studied worldwide. Until now, the biofuel that can be successfully produced by microbial fermentation is bioethanol: the global production of bioethanol in 2011 reached 84.6 billion liters, and 87 % of which were produced and used in the USA and Brazil (Renewable Fuels Association: 2012 Ethanol Industry Outlook, http://www.ethanolrfa.org). However, there are other candidates for biofuels. Hydrocarbon-based biofuels would be ideal as alternative fuels with several potential advantages (Antoni et al. 2007; Schirmer et al. 2010; Lennen et al. 2010). For the use of hydrocarbon as fuel, modification of the existing infrastructure, including engine and the distribution system, is not required. Hydrocarbons with saturated and longer carbon chains contain higher energy than ethanol or butanol, and its use can increase the average gas mileage of renewable fuels. Hydrocarbon has the further advantage of processing itself when it is produced by microbial fermentation: cost reduction and time saving by a simple separation process due to its immiscibility in water. ‘Biohydrocarbon’ is one of the next-generation biofuels, and its production by microbial fermentation is a new challenge. However, as with other fuels, hydrocarbons are toxic for bacteria. Therefore, improved bacterial tolerance to hydrocarbons by engineering is a main prerequisite for the microbial production of hydrocarbon.

The cell toxicity of an organic solvent, including hydrocarbons, is correlated with its low log*P*_ow_ (the logarithm of the partitioning coefficient of a solvent in a defined octanol–water mixture) that determines the degree of accumulation of the solvent in the cell membrane (Ramos et al. 2002; Sikkema et al. 1995). The accumulation of solvents in the membrane destroys the membrane barrier, increases permeability, impairs energy transduction by dissipation of the membrane potential, causes dysfunctions of membrane proteins such as transport and signaling, and finally results in cell death (Ramos et al. 2002; Sikkema et al. 1995).

The organic solvent tolerances (OSTs) of Gram-negative bacteria are closely related with the fatty acid composition of the cell membrane, cell envelop, multidrug efflux systems, osmoprotectant, and the stress response mechanism (Sikkema et al. 1995; Ramos et al. 2002; Goodarzi et al. 2010). However, it has been reported that the engineering of a similar target mechanism caused often contradictory results in tolerance (Ramos et al. 1997; Luo et al. 2009). For the engineering of OSTs, therefore, the selection of genes among targeting mechanisms is critical; OSTs depend also on the microorganisms and the properties of biofuels.

For targeted genetic engineering to improve tolerance, we investigated in this study 12 genes which participate in relevant mechanisms to tolerance. *n*-Hexane and cyclohexane were mainly used as organic solvents for testing. The hexanes are significant constituents of gasoline, and the cell toxicities of two hexanes are distinctly differentiated. We screened which targeting of the 12 genes confers high survival of bacteria under stress of solvents. The enhanced OSTs of single and double mutants of the genes *fadR* and *marR* were quantitatively investigated, and then the fatty acid composition and solvent accumulation in the OST strains were analyzed.

## Materials and methods

### Bacterial strains and growth conditions

The *Escherichia coli* strains and plasmids used in this study are listed in Table 1. Single gene knockout mutants were obtained from the National Institute of Genetics in Japan (Baba et al. 2006) or constructed according to Datsenko and Wanner (2000). The P1_kc_ transduction was used for the construction of a double-gene disruption mutant. All strains were cultivated in modified Luria–Bertani (LB) medium (LBGMg medium; Aono et al. 1991) containing 8 g/l Bacto peptone, 4 g/l yeast extract, 8 g/l NaCl, 1 g/l d-glucose, and 2.465 g/l MgSO_4_⋅7H_2_O. Cultures for the growth test were performed at 37 °C with 180 rpm in a shaking incubator. *n*-Hexane (Junsei), *n*-octane (Kanto Chemical), *n*-dodecane (Kanto Chemical), and cyclohexane (Sigma-Aldrich) were used for the OST test.

**Table 1:** *E*. *coli* strains, plasmids, and phage used in this study

	Relevant genotype	References
Strains		
BW25113	*lacI* ^q^ *rrnB*_*T14*_ Δ*lacZ*_*WJ16*_ *hsdR514* Δ*araBAD*_*AH33*_ Δ*rhaBAD*_*LD78*_	Baba et al. (2006)
JW0453	BW25113, but Δ*acrR*::*kan* ^R^	Baba et al. (2006)
JW0305	BW25113, but Δ*betI*::*kan* ^R^	Baba et al. (2006)
JW4093	BW25113, but Δ*cadB*::*kan* ^R^	Baba et al. (2006)
JW4094	BW25113, but Δ*cadC*::*kan* ^R^	Baba et al. (2006)
JW2779	BW25113, but Δ*gcvA*::*kan* ^R^	Baba et al. (2006)
JW3935	BW25113, but Δ*fabR*::*kan* ^R^	Baba et al. (2006)
JW1176	BW25113, but Δ*fadR*::*kan* ^R^	Baba et al. (2006)
JW5248	BW25113, but Δ*marR*::*kan* ^R^	Baba et al. (2006)
JW5249	BW25113, but Δ*marA*::*kan* ^R^	Baba et al. (2006)
JW4355	BW25113, but Δ*slt*::*kan* ^R^	Baba et al. (2006)
JW5670	BW25113, but Δ*yhiD*::*kan* ^R^	Baba et al. (2006)
LMB003	MG1655, but Δ*fabB*::*kan* ^R^	This study
LMB014	BW25113, but Δ*fadR*	This study
LMB015	BW25113, but Δ*fadR* Δ*marR*::*kan* ^R^	This study
Plasmids		
pCP20	FLP^+^ *λcI857 λp*_*R*_ *Rep* ^*ts*^ *cat bla*	Datsenko and Wanner (2000)
pKD46	*oriR101 repA101*(ts) *ara Bp*-*gam*-*bet*-*exo bl*	Datsenko and Wanner (2000)
pKD3	*oriRγ cat bla* Δ (*phoB*-*phoR*)*580 galU95* Δ*uidA3*::*pir* ^+^ Δ*endA*::FRT	Datsenko and Wanner (2000)
Phage		
P1_kc_		Miller (1992)

### Spot assay

Stains were grown with the LBGMg medium up to the late exponential phase and all cultures were resuspended with the same medium to an OD_600_ of 0.5. The bacterial suspensions were diluted in tenfold serial steps up to the 10^−6^ dilution stage. Of each diluted suspension, 5 μl was spotted on the LBGMg agar medium (approx. 20 ml medium) and overlaid with undiluted solvents *n*-hexane (4 ml), octane (4 ml), dodecane (4 ml), and *n*-hexane/cyclohexane mixture (1:1, *v*/*v*; total 2 ml). The growths were observed after 20 h of incubation at 30 °C. For the growth of mutants, 50 μg/ml kanamycin or 30 μg/ml chloramphenicol was contained in the medium.

### Pooled culture system and PCR confirmation

Each of 11 single gene mutants (*acrR*, *betI*, *cadB*, *cadC*, *gcvA*, *fabR*, *fadR*, *marR*, *marA*, *slt*, and *yhiD*) was grown with the LBGMg medium containing 50 μg/ml kanamycin at 37 °C to the late exponential phase (OD_600_ = 1.5–2.0). Bacterial cells were harvested by centrifugation (4,000 rpm, 10 min at 4 °C) and resuspended with LBGMg medium to OD_600_ = 0.5. The same volumes of the 11 bacterial suspensions were mixed and named “the pooled culture” (Dunlop et al. 2011). The bacterial cells from the pooled culture were successively cultivated nine times at 37 °C in 30 ml LBGMg medium with or without organic solvents. As solvent stresses, 10 % of *n*-hexane (*v*/*v*) or 2 % of *n*-hexane/cyclohexane mixture (1:1, *v*/*v*) was added to the culture. Each cultivation lasted for 12 h; the culture was immediately inoculated (1 %, *v*/*v*) into fresh medium. During the successive cultivation, the relative quantitative change of growth in the culture was monitored by PCR. Total genomic DNA from the pooled culture (zeroth), the third, fifth, seventh, and ninth successive cultures was isolated with a genomic DNA prep kit (Solgent Co., South Korea) and used to template for PCR. The specific sequence of each genomic DNA was amplified with each primer set (Cosmo GENEtech., South Korea). All reverse primers “Δ(*genes*)_pooled_rev” were paired with “kan2” (5′-CGG TGC CCT GAA TGA ACT GC-3′) as a forward pair. In order to get similar PCR efficiency for the 11 genes, all sets of primers were designed to amplify DNA of approx. 1 kb. The four primers used in Fig. 2 are as follows: Δ*acrR*_pooled_rev (5′-GCG TTC GGG CTG CGT CTG TA-3′), Δ*fabR*_pooled_rev (5′-CGG TTA CGC ATC TTC GCG CC-3′), Δ*fadR*_pooled_rev (5′-CCG GTT CCG ACT GGC TGG AA-3′), and Δ*marR*_pooled_rev (5′-GGT TTG TTC CGC AAC GCC CT-3′). PCR was performed with REDTaq®ReadyMix^TM^ (Sigma-Aldrich) in 25 cycles using My Cycler^TM^ (Bio-Rad).

### Growth and cell viability test

For the growth and viability test, overnight subcultures of BW25113, JW1176, JW5248, and LMB015 were inoculated 1 % (*v*/*v*) in fresh LBGMg medium. The solvent was added 1 h after inoculation (OD_600_ of about 0.2). The microbial cell viability assay was performed with a Microbial Viability Assay Kit-WST (Dojindo, Japan). The electron mediator (2-methyl-1,4-naphthoquinone) receives electrons from viable bacterial cells and transfers the electrons to the WST reagent. The absorbance of the reduced WST reagent was measured with a microplate reader (BioTek, USA) at 450 nm (Tsukatani et al. 2008).

### Analysis of cellular fatty acid composition

Each strain of BW25113, JW1176, JW5248, and LMB015 was successively cultivated ten times in LBGMg medium at 37 °C with or without *n*-hexane 10 % (*v*/*v*). Each cultivation ran 12 h; the culture was immediately inoculated (1 %, *v*/*v*) into fresh medium. The tenth culture was harvested at OD_600_ = 4 and washed with fresh medium with centrifugation at 4,000 rpm and RT for 10 min. Fatty acid methyl esters were extracted from a cell pellet (40–50 mg) with hexane/methyl *tert*-butyl ether (1:1, *v*/*v*). The fatty acid composition was analyzed by GC (HP6890, Agilent Technologies Inc., USA) with a cross-linked methyl siloxane column (HP-1, 30 m × 0.320 mm × 0.25 μm; Agilent Technologies Inc.) and a flame ionization detector (FID) in the Korean Culture Center of Microorganisms (Seoul, South Korea; Miller 1982). The column temperature was raised at a rate of 5 °C/min from 170 to 270 °C.

### Measurement of intracellular *n*-hexane accumulation

The bacterial cells were inoculated 1 % (*v*/*v*) in 10 ml LBGMg medium and cultivated to OD_600_ = 1.5–2. After 1 h of incubation with *n*-hexane (10 %, *v*/*v*), the *n*-hexane was extracted from *E*. *coli* cells using a modified method by Tsukagoshi and Aono (2000). From 8 ml of the harvested culture, the solvent layer was first removed and then the medium layer was discarded. The cell pellet was resuspended in 2 ml of 0.9 % NaCl–10 mM MgSO_4_ and mixed with the same volume of CHCl_3_. *n*-Hexane was extracted two times by vortexing (30 s) with an interval of vigorous shaking for 10 min. After centrifugation, the CHCl_3_ layer was transferred for gas chromatography.

The amount of *n*-hexane in the CHCl_3_ extract was measured using a GC (GC6850N, Agilent Technologies Inc.) with a wax column (30 m × 0.32 mm × 0.25 μm; Supelco, USA) and detected with a FID. Of the extract, 1 μl was injected; the inlet temperature was 230 °C. The column was eluted with N_2_ gas at a flow rate of 35 ml/min. The oven temperature holds 100 °C for 5 min and then increases to 320 °C at 5 °C/min. The intracellular concentration of *n*-hexane was calculated with the cell dry weight of each strain. An OD_600_ = 1 of strains BW25113, JW1176, JW5248, and LMB015 corresponded to 460, 660, 650, and 590 μg cell dry weight per milliliter, respectively.

## Results

### Selection and screening of target genes: deletion of *fadR* and *marR* increased organic solvent tolerances in the spot assay

The bacterial OSTs are influenced by complex cellular mechanisms. Organic solvents can be inserted in the membrane, destroy the biological functions of the membrane, and disturb proteins in the membrane or in the cytosol, which hinders diverse cellular reactions, including energy processing, solute transport, and signal transduction (Sikkema et al. 1995; Ramos et al. 2002). Overall, they break down cellular homeostasis and induce stress responses. We selected a total of 12 target genes (Table 2) whose manipulation may prevent the toxic effect of biofuel in the form of hydrocarbon: *fabR*, *fadR*, *fabB*, *acrR*, *marA*, *marR*, *betI*, *gcvA*, *slt*, *cadB*, *cadC*, and *yhiD*. The *fabR* and *fadR* genes regulate fatty acid biosynthesis, and *fabB* is directly involved in fatty acid synthesis (Campbell and Cronan 2001). The genes *acrR*, *marA*, and *marR* are involved in the regulation of multi-drug resistance (MDR) (Ruiz and Levy 2010). The *betI* and *gcvA* genes are related to the cellular osmoprotectants (Goodarzi et al. 2010). The *slt* is involved in the lysis of the peptidoglycan during cell growth (Vollmer and Bertsche 2008). The acid stress response genes *cadB*, *cadC*, and *yhiD* are recently reported as putative genes related to alcohol tolerances (Goodarzi et al. 2010).

**Table 2:** Results of the spot assay with the targeted 12 candidate genes

Categories of mechanism	Genes	Tolerances^a^
Fatty acid synthesis	*fabR*	−
	*fadR*	+
	*fabB*	*n*
Multi-drug resistance	*acrR*	−
	*marA*	−
	*marR*	+
Cellular osmoprotectant	*betI*	*n*
	*gcvA*	−
Peptidoglycan production	*Slt*	*n*
Acid stress response	*cadB*	−
	*cadC*	−
	*yhiD*	*n*

The assay was performed in the presence of 10 % *n*-hexane, and the colony formation of the mutant strains were compared to the wild-type strain

^a^Change of tolerance was indicated as increased (+), decreased (−), or unchanged (*n*) growth in colony formation of knockout mutants compared to the wild-type strain

**Fig. 1:**
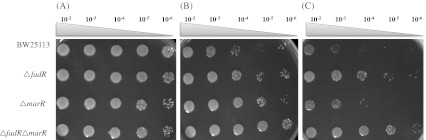
Spot assay with *E*. *coli* strains on LBGMg agar medium overlaid without solvent (**a**), with 4 ml of *n*-hexane (**b**), and with 2 ml of *n*-hexane/cyclohexane mixture (1:1, *v*/*v*) (**c**). Tenfold serial dilutions of cell suspensions from 10^−2^ to 10^−6^ were spotted and grown at 30 °C for 20 h

The spot assay of the 12 target mutants with *n*-hexane or *n*-hexane/cyclohexane mixture showed that the tolerances increased in the Δ*marR* mutant (JW5248) and the Δ*fadR* mutant (JW1176) significantly (Fig. 1). The Δ*marR* and Δ*fadR* mutants formed colonies when more diluted cells were spotted. All the other tested ten single mutants grew similarly or weakened compared with the wild-type strain BW25113 (data not shown). The Δ*fadR* Δ*marR* double mutant (LMB015) showed more significantly improved tolerances than each Δ*fadR* or Δ*marR* single mutant (Fig. 1). The growth of the double mutant with spotting of 10^3^ more diluted cells was comparable to that of the wild-type (BW25113) cell in the presence of *n*-hexane and *n*-hexane/cyclohexane mixture. The wild-type strain (BW25113) could hardly grow especially in the presence of the solvent, especially with cyclohexane. The growth of all the tested 12 strains showed no differences in the presence of *n*-octane or *n*-dodecane up to 20 % (*v*/*v*; data not shown).

### The Δ*marR* mutant became the dominant strain in pooled culture during competitive growth with *n*-hexane and cyclohexane

**Fig. 2:**
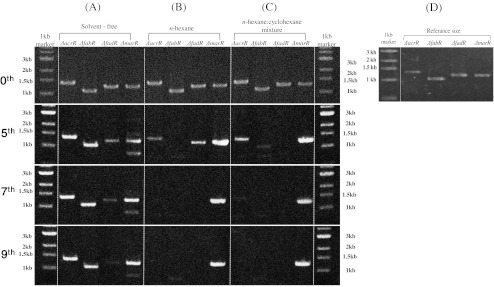
Monitoring of change of bacterial population by PCR. Total genomic DNAs were isolated from the zeroth, third, fifth, seventh, and ninth successive cultivations started from pooled culture and used as the template for PCR. The successive cultivation was performed without any solvents (**a**), with *n*-hexnae (10 %, *v*/*v*) (**b**), and with *n*-hexane/cyclohexane mixture (1:1, 2 %, *v*/*v*) (**c**). The reference PCR products corresponded to the strains are shown in (**d**)

The pooled culture consisting of 11 different single mutants (Δ*fabR*, Δ*fadR*, Δ*acrR*, Δ*marA*, Δ*marR*, Δ*betI*, Δ*gcvA*, Δ*slt*, Δ*cadB*, Δ*cadC*, and Δ*yhiD*), each with the same cell number, was cultivated with solvents nine times successively by immediate inocula. Comparative changes of the bacterial populations during successive solvent trials were monitored with PCR. Changes of the four regulator mutants (Δ*acrR*, Δ*fabR*, Δ*fadR*, and *ΔmarR*) are shown in Fig. 2. Without solvents, the three mutants Δ*acrR*, Δ*fabR*, and Δ*marR* were maintained during successive cultivation, but the mutant Δ*fadR* was gradually reduced and nearly disappeared in the ninth cultivation (Fig. 2a). During cultivation with *n*-hexane, the Δ*acrR* and Δ*fadR* mutants remained until the fifth cultivation, but are lost in the seventh culture (Fig. 2b). A portion of the Δ*fabR* mutant was significantly diminished in the third culture (data not shown) and disappeared in the fifth. On the other hand, the Δ*marR* mutant survived in culture and thrived as the only strain in the seventh and the ninth culture.

With the *n*-hexane/cyclohexane mixture, the Δ*marR* mutant survived to the end, the Δ*acrR* mutant remained until the fifth culture, and the Δ*fabR* mutant nearly disappeared in the fifth, which are comparable to the solvent trial with *n*-hexane (Fig. 2c). But the Δ*fadR* mutant was lost earlier compared to that with *n*-hexane. The other tested seven mutants (Δ*marA*, Δ*betI*, Δ*gcvA*, Δ*slt*, Δ*cadB*, Δ*cadC*, and Δ*yhiD*) disappeared already in the third cultivation in both solvent trials (data not shown). Consequently, the Δ*marR* mutant became the dominant and was the only surviving strain in the pooled culture during competitive successive cultivations with *n*-hexane and the *n*-hexane/cyclohexane mixture.

### The still more improved solvent tolerance of the Δ*fadR* Δ*marR* double mutant LMB015

**Fig. 3:**
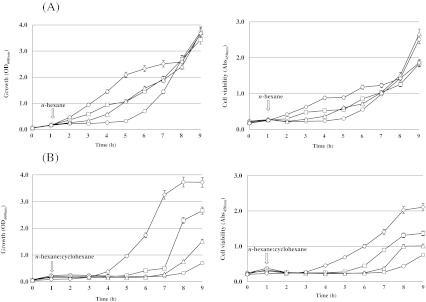
Growth and cell viability of bacterial strains with *n*-hexane (10 %, *v*/*v*) (**a**) and *n*-hexane/cyclohexane mixture (1:1, 2 %, *v*/*v*) (**b**). Solvent was added to the culture 1 h after the start of cultivation. Wild-type BW25113 (*circle*), *ΔfadR* mutant JW1176 (*square*), *ΔmarR* mutant JW5248 (*triangle*), and *ΔfadR ΔmarR* mutant LMB015 (*diamond*)

The growth and cell viability of Δ*fadR* and/or the Δ*marR* mutant were quantitatively studied in liquid culture. Under *n*-hexane stress, the single mutants, JW1176 (Δ*fadR*) and JW5248 (Δ*marR*), grew significantly better than the wild-type strain (Fig. 3a). The tolerance of the Δ*fadR* Δ*marR* double mutant LMB015 was enhanced even more remarkably than that of each single mutant. After 4–6 h of incubation with *n*-hexane (10 %, *v*/*v*), the cell number and the viability of the double mutant are nearly twice those of the single mutant. At 5 h, the OD_600_ of strains were in the following order: LMB015 (Δ*fadR* Δ*marR*, OD_600_ = 2.1) > JW1176 (Δ*fadR*, OD_600_ = 1.1), JW5248 (Δ*marR*, OD_600_ = 1.1) > BW25113 (wt, OD_600_ = 0.3). The values of cell viability corresponded to OD_600_.

The distinguished enhanced tolerance of double mutants was showed in the growth experiment with *n*-hexane/cyclohexane mixture (2 %, 1:1, *v*/*v*) more significantly (Fig. 3b). Until 7 h of incubation with a solvent, only the double mutant LMB015 (Δ*fadR* Δ*marR*) could grow well and reached an OD_600_ of 3.3. In contrast, the growth of the other strains—JW1176 (Δ*fadR*, OD_600_ = 0.50), JW5248 (Δ*marR*, OD_600_ = 0.28), and BW25113 (wt, OD_600_ = 0.14)—remained in the lag phase. The two single mutants (Δ*fadR* and Δ*marR*) showed growth only from 8 h. The viability test exhibited the same tendency in the growth curve.

### The elevated contents of saturated fatty acid in the Δ*fad* mutant

The cellular fatty acid composition of bacteria was investigated in relation to tolerance between strains and their change during solvent stress. After ten times successive solvent trials, total cellular fatty acids were analyzed using a gas chromatograph (Table 3). Most of the differences in the fatty acid composition between strains lay in the content ratios of palmitic acid (C_16:0_) and vaccenic acid (C_18:1_). Without *n*-hexane stress, the palmitic acid ratios in JW1176 (Δ*fadR*, 39.6 %) and LMB015 (Δ*fadR* Δ*marR*, 39.7 %), in which the gene *fadR* is deleted, were about 16 % higher than that in BW25113 (wt, 23.7 %). The contents of vaccenic acid in both mutants (8.1 and 5.5 %) were 14.1 and 16.7 % lower than in BW25113 (wt, 22.2 %). Between BW25113 (wt) and JW5248 (Δ*marR*), there was no significant difference in the fatty acid composition. The total ratios of saturated fatty acid to unsaturated fatty acid (SFA/UFA) were 8.4 in JW1176 (Δ*fadR*) and 13.3 in LMB015 (*ΔfadR ΔmarR*), but only 2.5 in BW25113 (wt) and 2.2 in JW5248 (Δ*marR*). The regulator FadR functions as an activator for key enzymes, *fabA* and *fabB*, in unsaturated fatty acid synthesis (Campbell and Cronan 2001). In JW1176 (Δ*fadR*) and LMB015 (Δ*fadR* Δ*marR*), the biosynthesis of unsaturated fatty acid could not be activated due to deficiency of the activator FadR, thereby elevating the content of the saturated fatty acid, palmitic acid.

**Table 3:** Cellular fatty acid composition in four strains analyzed using GC after successive cultivations without (−) and with (+) *n*-hexane

Fatty acids		BW25113		JW1176 (*ΔfadR*)		JW5248 (*ΔmarR*)		LMB015 (*ΔfadR ΔmarR*)	
		−	+	−	+	−	+	−	+
SFA	12:0	5.5	3.5	4.7	4.6	5.2	4.1	5.1	5.3
	14:0	6.9	7.2	7.2	9.4	7.1	6.7	11.4	13.0
	16:0	23.7	32.8	39.6	44.5	24.0	29.8	39.7	40.6
	17:0 Cyclo	14.7	10.2	15.2	16.6	12.9	15.5	15.0	15.7
	18:0	0.4	0.5	0.4	0.5	0.4	0.6	0.3	0.5
	19:0 Cyclo	4.1	2.3	1.3	1.6	3.5	5.0	1.2	1.2
	Others	0.3	0.5	0.3	0.4	0.3	0.5	0.3	1.2
UFA	16:1	0.4	0.2	0.1	nd	0.6	0.5	nd	nd
	18:1	22.2	20.3	8.1	4.9	23.9	21.0	5.5	4.8
	Others	nd	nd	nd	nd	nd	0.1	nd	0.1
Unknown FA		22.1	22.5	23.3	17.5	22.3	16.7	21.5	17.9
Total		100.3	100.0	100.2	100.0	100.2	100.5	100.0	100.3
Total SFA		55.6	57.0	68.7	77.6	53.4	62.2	73.0	77.5
Total UFA		22.6	20.5	8.2	4.8	24.5	21.6	5.5	4.9
SFA/UFA ratio		2.5	2.8	8.4	16.2	2.2	2.9	13.3	15.8

The amounts of fatty acids were given as the ratio (in percent) to the total intracellular fatty acid. The ratios given are the mean of double independent samples

*SFA* saturated fatty acid, *UFA* unsaturated fatty acid, *nd* not detected

During successive *n*-hexane stresses, the total SFA content including palmitic acid was increased to some extent in all strains, and the total UFA was decreased accordingly. The total ratio of SFA/UFA in both *fadR* mutants reached about 16 after *n*-hexane stress: 16.2 in JW1176 (Δ*fadR*) and 15.8 in LMB015 (Δ*fadR* Δ*marR*). In BW25113 (wt) and JW5248 (Δ*marR*), the ratios of SFA/UFA were 2.8 and 2.9, respectively. The high ratio of saturated fatty acid in the membrane showed the strength of the bacteria against the solvent in the spot assay (Fig. 1) and in the growth and viability test (Fig. 3).

The OSTs caused by the deletion of *fadR* are attributed to the elevated ratio of SFA/UFA, but the tolerance in the Δ*marR* mutant is not directly related to the SFA/UFA ratio.

### *n*-Hexane was less accumulated in OST strains than in the wild-type strain

The amount of remaining intracellular solvent is a criterion of solvent tolerance. Intracellular *n*-hexane from strains after cultivation with *n*-hexane was investigated using GC. The intracellular *n*-hexane levels of the mutants were significantly lower than that of the wild-type (Table 4). The intracellular *n*-hexane amounts of each mutant—JW1176 (Δ*fadR*, 513 ng/mg cell dry weight (cdw)), JW5248 (Δ*marR*, 524 ng/mg cdw), and LMB015 (*ΔfadR ΔmarR*, 588 ng/mg cdw)—were 46, 45, and 38 % less than that of BW25113 (wild-type, 953 ng/mg cdw). The high content of SFA in the cell membrane of the Δ*fadR* mutant could inhibit the penetration of *n*-hexane, and a reinforced MDR in the Δ*marR* mutant could pump out the imported *n*-hexane. The intracellular *n*-hexane level of the *ΔfadR ΔmarR* double mutant was nearly the same as that of each single mutant.

**Table 4:** Analysis of intracellular accumulation of *n*-hexane using GC

Strain	Amount of *n*-hexane (μg/g cell dry weight)
BW25113 (wild-type)	953 ± 22
JW1176 (*ΔfadR*)	513 ± 46
JW5248 (*ΔmarR*)	524 ± 37
LMB015 (*ΔfadR ΔmarR*)	588 ± 42

Reference data from GC analysis without *n*-hexane were provided as blank values. Shown are the means from at least three independent experiments

## Discussion

Tolerance acquisition of microorganisms is a breakthrough to promote the biotechnological production of biofuels. In this study, we showed a highly increased solvent tolerance of the *ΔfadR ΔmarR* double mutant in which two kinds of physiological functions of bacteria were simultaneously changed due to the deletion of regulator genes in their respective pathway: fatty acid synthesis and multidrug resistance (MDR).

The fatty acid composition of the membrane determines directly the membrane permeability and influences indirectly the activities of membrane proteins which function in solute transport, protein–protein interaction, and energy production. Biofuels, in the form of organic solvents, are inserted in the membrane or passed through the membrane, by which the functions of the cell membrane are collapsed. The first point in engineering to enhance tolerance was preventing the penetration of the solvents into the membrane. The tightening membrane would be achieved through an increased ratio of saturated fatty acid (SFA) to unsaturated fatty acid (UFA). We showed that the increased SFA content in the *fadR* mutant (Table 3) made the membrane less permeable to the solvent, resulting in a lower amount of intracellular *n*-hexane (Table 4) and, accordingly, enhanced tolerance to *n*-hexane and *n*-hexane/cyclohexane mixture (Figs. 1 and 3). The bifunctional regulator FadR plays roles in modulating lipid metabolism in *E*. *coli*: it represses the *fad* (fatty acid degradation) regulon (Cronan and Subrahmanyam 1998; Campbell and Cronan 2002; Iram and Cronan 2005; Feng and Cronan 2009) while it activates UFA biosynthesis (Henry and Cronan 1991; Campbell and Cronan 2001). FadR is responsible for the maximal expression of two key enzymes—FabA (3-hydroxydecanoyl-ACP dehydratase) and FabB (β-ketoacyl-ACP synthase I)—in UFA biosynthesis, whereas FabR (fatty acid biosynthesis repressor) functions as a repressor for the two enzymes. It was reported that an excess of FabA leads to an increased SFA content (Luo et al. 2009), and it could be postulated that the balance of FabA and FabB plays a role to determining the SFA/UFA ratio. The lack of activator FadR led to enhanced tolerance to solvents due to the high ratio of SFA/UFA in this study, but the *fabB* mutant did not show increased survival (Table 2).

The second point in engineering bacteria was the establishment of a more rapid solvent–efflux system by the increased AcrAB-TolC efflux pump which excretes many antimicrobial drugs and organic solvents (White et al. 1997; Okusu et al. 1996). The overexpression of the global regulator MarA in the *marR* mutant gave bacteria enhanced tolerance to *n*-hexane and/or cyclohexane (Asako et al. 1997; Table 2 and Figs. 1, 2, and 3). The lack of MarR leads to the overproduction of MarA, by which the global network “MarA-mediated MDR” is turned on (Barbosa and Levy 2000; Alekshun and Levy 2004). Operon *marRAB* encodes repressor MarR, activator MarA, and putative protein MarB. The expression of the operon is regulated by multiple transcription factors including its own products, MarR and MarA, with their auto-regulative activities. Representative MarA-mediated upregulated genes are *acrA*, *acrB*, and *tolC* for AcrAB-TolC efflux, but also the expressions of over 60 chromosomal genes were regulated to elevate MDR at once (Ruiz and Levy 2010). We also tested the solvent tolerance of the *acrR*-deleted mutant in which the expression of the *acrAB* operon increased due to the lack of repressor AcrR, but the *acrR* mutant showed no enhanced tolerance (Table 2). Deletion of only the local regulator AcrR may not be sufficient to produce efflux pump, if the expression of TolC was limited.

The *ΔfadR ΔmarR* mutant, having a tightened membrane due to the deletion of *fadR*, became even more solvent-tolerant when MDR was strengthened by additional deletion of *marR* (Figs. 1 and 3). The ratio of SFA/UFA in the *ΔfadR ΔmarR* double mutant was nearly unchanged (13.3–15.8) during cultivation with *n*-hexane compared with the *fadR* mutant (8.4–16.2; Table 3). Maintaining a high ratio of SFA/UFA in the presence of *n*-hexane can be considered a factor for having distinguished tolerance of the double mutant (Figs. 1 and 3). The unchanged ratio of SFA/UFA in the *ΔfadR ΔmarR* mutant is due to MarA decreasing the gene expression of *fabB* about 2.9-fold, according to Barbosa and Levy (2000). In the double mutant, not only the two mechanisms—fatty acid composition and AcrAB-TolC efflux pump—but also hundreds of genes were influenced by a change of two global regulators: deletion of FadR and overproduction of MarA. These synergy effects on enhancing tolerance in the *ΔfadR ΔmarR* double mutant will be investigated in more detail in a further study.
